# Sociodemographic determinants of health care in heart failure mortality in Mexico, 019 and 2023

**DOI:** 10.1590/1980-549720260019.2

**Published:** 2026-04-20

**Authors:** Julio Cesar Campuzano, Jorge Martin Rodríguez, Pablo Enrique Chaparro, Diana Carolina Urrego, Anaid Hernández

**Affiliations:** I Instituto Nacional de Salud Pública de México - Cuernavaca, Morelos, Mexico.; II Pontificia Universidad Javeriana, Instituto de Salud Pública - Bogotá D.C., Colombia.; III Observatorio Nacional de Salud - Bogotá D.C., Colombia.; IV Ministerio de Salud y Protección Social - Bogotá, Colombia.; V Universidad Latinoamericana - Cuernavaca, Morelos, Mexico.

**Keywords:** Heart failure, Mortality, Socioeconomic factors, Social security, Mexico

## Abstract

**Objective:**

To analyze sociodemographic determinants associated with medical care before death from heart failure (HF) in Mexico, comparing 2019 and 2023.

**Methods:**

A retrospective cross-sectional analytical study was conducted using national mortality records from the General Directorate of Epidemiology (DGE). HF deaths (ICD-10: I50.0-I50.9) from 2019 and 2023 were selected, representing pre- and post-pandemic (COVID-19) contexts. The variables included gender, age, education, marital status, health insurance coverage, area of residence, region, place of death, and year of occurrence. Multiple logistic regression was applied to estimate odds ratios (OR) with 95% confidence intervals (95% CI).

**Results:**

In the 2019-2023 period, 13,510 HF deaths were recorded (6,077 in 2019 and 7,433 in 2023). The probability of receiving medical care before death was 22% higher in 2023 compared to 2019 (OR 1.22; 95%CI 1.10-1.36). Medical care was more likely among individuals aged ≥80 years old, with higher educational levels, living in urban areas, and with health insurance coverage. Being male, lacking health coverage, and dying at home were associated with a lower probability of receiving medical care. The interaction between health insurance coverage and place of death revealed reduced access to medical care among individuals without social security who died at home.

**Conclusions:**

Substantial gaps in access to medical care persist, associated with structural and social inequalities. The post-pandemic recovery of coverage indicates a partial strengthening of the health system. Strengthening primary health care, expanding effective universal coverage, and developing strategies targeting vulnerable groups are recommended.

## INTRODUCTION

Heart failure (HF) is a clinical syndrome characterized by signs and symptoms resulting from structural and/or functional cardiac abnormalities that impair the heart’s ability to pump sufficient blood to meet the body’s metabolic demands^1^. The principal risk factors for the development of HF include coronary heart disease, hypertension, diabetes mellitus, obesity, a family history of cardiovascular disease, chronic lung disease, chronic inflammation or infection, metabolic disorders, exposure to cardiotoxic agents, cocaine use, anthracycline therapy in oncology, and alcohol abuse^2^.

Since the late 20^th^ century, HF has been recognized as an epidemic due to the exponential rise in hospitalizations attributable to this condition^3^. In 2002, it was estimated that approximately 6.5 million individuals in Europe, 5 million in the United States, and 2.4 million in Japan were living with the disease^4^. This increase has been associated with population aging^5^, as well as with a higher prevalence of comorbidities, greater physical disability, and an increased incidence of adverse events, all of which contribute to higher healthcare utilization and mortality. Consequently, HF has emerged as a major public health concern^6^.

Cardiovascular diseases are currently the second leading cause of death worldwide, following cancer^7^, and represent the leading cause of death in Mexico. According to estimates from the Global Burden of Disease 2023 study, more than 64 million individuals are living with HF globally, with an age-adjusted prevalence of 831 cases per 100,000 inhabitants^8^. The greatest burden is concentrated in low- and middle-income countries, where incidence and mortality rates remain high due to limited access to healthcare services and a higher prevalence of metabolic risk factors. In contrast, high-income countries have demonstrated a sustained reduction in HF-related mortality, attributable to the expansion of evidence-based pharmacological therapies and the strengthening of primary cardiovascular care^9^.

However, although current advances in the treatment of HF have improved patients’ quality of life and survival, an increase in comorbidities, disability, and functional dependence has also been observed, leading to higher healthcare system costs. This phenomenon is related not only to increased longevity but also to population aging, which is recognized as one of the main risk factors for the development of HF^10^.

Although current technologies have made it possible to stabilize or even reduce the incidence of HF in older adults in some populations, opposite trends have been observed in younger individuals, possibly related to the increased prevalence of other pathologies, such as obesity^11^. Additionally, it has been demonstrated that characteristics such as race influence the incidence of HF before the age of 50 years, with higher frequency observed in Black individuals compared with White individuals^12^.

Despite the importance of this disease, estimates of the incidence of HF and their temporal trends remain scarce and inconsistent, even in high-income countries^13^. In Mexico, although precise data are not available, it is estimated that 750,000 individuals suffer from HF, with approximately 75,000 new cases occurring each year^14^. This situation is further aggravated by structural inequalities in access to healthcare services.

The health system in Mexico comprises public and private sectors that operate in a complementary manner, although with marked inequalities. The public sector includes social security institutions - the Mexican Social Security Institute (*Instituto Mexicano del Seguro Social* - IMSS), the Institute for Social Security and Services for State Workers (*Instituto de Seguridad y Servicios Sociales de los Trabajadores del Estado* - ISSSTE), *Petróleos Mexicanos* (PEMEX), the Ministry of National Defense (*Secretaría de la Defensa Nacional* - SEDENA), and the Ministry of the Navy (*Secretaría de Marina* - SEMAR) - which provide coverage to approximately 52% of the population^15^. Meanwhile, the system for the care of the uninsured population, formerly known as *Seguro Popular* and, since 2023, the Mexican Social Security Institute for Well-being (*Instituto Mexicano de Seguridad Social Bienestar* - IMSS Bienestar), covers approximately 35% of the population, while the remaining 13% rely on private services or have limited effective access to healthcare^16^. These structural differences in affiliation and resource availability affect equity in healthcare delivery, particularly in the management of chronic diseases such as HF^17^.

The magnitude of this disease, its impact on mortality, and the high costs associated with its treatment highlight its relevance to public health. In this context, the study of mortality related to HF represents an essential tool for health planning, allowing the identification of inequalities, the prioritization of resources, and the formulation of evidence-based public health policies^18^.

Therefore, the objective of this study was to perform a comparative analysis of the sociodemographic determinants related to medical care among individuals who died from HF in Mexico during the years 2019 and 2023.

## METHODS

### Design and information sources

A retrospective, cross-sectional study with an analytical approach was conducted using individual death records obtained from the General Directorate of Epidemiology (*Dirección General de Epidemiología* - DGE) of Mexico. Deaths in which the underlying cause was HF were selected using codes I50.0 to I50.9 of the International Classification of Diseases, Tenth Revision (ICD-10).

The years 2019 and 2023 were selected to represent the periods before and after the COVID-19 pandemic, thereby avoiding potential bias from the intervening years (2020-2022), during which alterations in medical care delivery and in death certification processes were observed.

### Place and area of study

The study was conducted nationwide in Mexico, covering all 32 federal entities. The country has an area of 1.96 million km^2^ and an estimated population of 129 million inhabitants in 2023. The national Human Development Index (HDI) was 0.78, and the per capita income was US$12,900. The Central and Northern regions have the most extensive healthcare infrastructure, whereas the South and Southeast regions have historically shown lower coverage and quality of healthcare services^19^.

### Data source

Mortality data were obtained from the Epidemiological and Statistical Subsystem of Deaths (*Subsistema Epidemiológico y Estadístico de Defunciones* - SEED), administered by the DGE. This database is composed of death certificates processed by the Civil Registry and the National Institute of Statistics and Geography (*Instituto Nacional de Estadística y Geografía* - INEGI), with an estimated national coverage of 98%^20^. Previous studies have documented underreporting of less than 2%, with slight regional variations, mainly in southern states.

### Variables and information processing

The dependent variable was “medical assistance prior to death” (yes/no). Independent variables included state; geographic region (North, Central-North, Central, South, and Southeast); gender; age (under 60 years; 60 to 80 years; and over 80 years); month of death (December-January and other months); educational level (no schooling; primary/secondary education; and high school education or higher); marital status (married/other); health insurance coverage (with or without coverage); area of residence (urban or rural); year of death (2023/2019); speaks indigenous language (yes/no); place of death (home or other place); and birth cohort.

The dependent variable “medical assistance prior to death” demonstrated a completeness rate of 97.6%, which supports the validity of the analyses performed.

### Information analysis

Mortality rates per 100,000 inhabitants were calculated, stratified by age range and gender, with their respective 95% confidence intervals. The rates were estimated based on deaths registered by the DGE and population projections provided by the National Population Council (*Consejo Nacional de Población* - CONAPO) and INEGI.

### Bivariate analysis

The associations between medical care and categorical variables were explored using the chi-square test. For continuous variables, such as age, the Student’s *t*-test for independent samples was applied. Variables with *p*<0.05 in the bivariate analysis were included in the multiple logistic regression model.

### Multiple analysis

Based on the results of the cross-sectional design, a binary logistic regression model was applied to estimate odds ratios (ORs), 95% confidence intervals (95% CIs), and *p*-values. Categorical variables were transformed into dummy variables by excluding a reference category to avoid multicollinearity. The interaction between health insurance status and place of death was evaluated using an additive term (dh#soc) to identify combined effects on the probability of having received medical care prior to death.

The model was estimated using maximum likelihood estimation with the *Logit* link function from the *statsmodels* library in Python, supplemented with *pandas* and *numpy*. A robust variance-covariance matrix estimator (*cov_type*=’HC0’) was used to control for heteroscedasticity. ORs and their respective 95% CIs were reported.

### Ethical considerations

This study was classified as low risk, as it exclusively used anonymized, publicly available secondary databases. According to Article 17 of the Regulations of the General Health Law on Health Research, studies that use publicly available and anonymized data are considered low risk and are exempt from ethics committee review^21^.

## Data availability statement:

The full dataset supporting the results of this study is available from the corresponding author upon reasonable request.

## RESULTS

**Figure 1.**
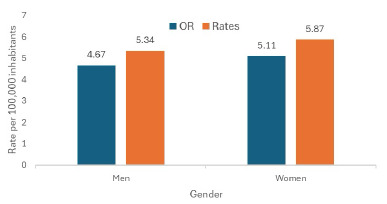
Comparison of mortality rates due to heart failure in men and women. Mexico, 2019 and 2023.

In Mexico, 13,510 deaths due to HF were registered between 2019 and 2023, of which 6,077 (45%) occurred in 2019 and 7,433 (55%) in 2023. The mortality rate per 100,000 inhabitants was higher in 2023 (5.61) than in 2019 (4.89), and was slightly higher among women than among men in both years (Figure 1).

In men, the rate increased from 4.67 to 5.34 per 100,000 inhabitants, while in women it increased from 5.11 to 5.87 per 100,000 inhabitants, reflecting a sustained rise in both genders, although more pronounced among men.

Table 1 summarizes the sociodemographic characteristics of individuals who died from HF according to medical care received prior to death. Overall, the majority of individuals received medical care before death. Women showed a higher proportion of receiving care (86.7%) than men (84.4%). Regarding age, the group aged 80 years and older accounted for the largest number of deaths (97%), and 15.1% of individuals in this age group did not receive medical care. By region, the central region of the country registered the highest proportion of individuals receiving care (92.2%), whereas the North and Southeast regions showed the lowest proportions of care received (19.2% and 19.3% without care, respectively).

Medical care was more frequent among individuals residing in urban areas (87.9%) than among those in rural areas (77.6%), as well as among married individuals (88.8%) compared with unmarried individuals (84.2%) (Table 1).

**Table 1.** Characteristics of individuals who died from heart failure according to sociodemographic aspects and medical care received. Mexico, 2019 and 2023.

Characteristic	Category	Received medical care (%)	Did not receive medical care (%)	95%CI
Gender	Male	84.4	15.6	83.2-85.6
	Female	86.7	13.3	85.5-87.9
Age (years)	<60	78.3	21.7	76.8-79.8
	60-79	85.2	14.8	84.1-86.3
	≥80	97.0	3.0	96.5-97.5
Education	No schooling	82.6	17.4	81.1-84.1
	Primary/secondary	88.9	11.1	87.8-90.0
	High school or higher	91.5	8.5	90.1-92.9
Marital status	Married	88.8	11.2	87.6-90.0
	Other	84.2	15.8	82.9-85.5
Health insurance coverage	With coverage	92.3	7.7	91.1-93.5
	Without coverage	78.4	21.6	77.0-79.8
Month of death	December-January	83.1	16.9	81.8-.84.5
	Other months	86.3	13.7	85.7-.86.9
Place of death	Home	80.6	19.4	79.6-81.5
	Other locations	90.9	9.1	90.2-91.6
Area of residence	Urban	87.9	12.1	86.5-89.3
	Rural	77.6	22.4	75.8-79.4
Region	North	80.8	19.2	78.9-82.7
	Central-North	89.1	10.9	87.8-90.4
	Central	92.2	7.8	91.0-93.4
	South	86.7	13.3	85.2-88.2
	Southeast	80.7	19.3	78.9-82.5
Year of death	2019	84.5	15.5	83.2-85.8
	2023	86.8	13.2	85.6-88.0

95%CI: 95% confidence interval.

The multiple logistic regression model (Table 2) showed that individuals who died in 2023 were 22% more likely to have received medical care than those who died in 2019 (OR 1.22; 95%CI 1.10-1.36). Men were 20% less likely to have received medical care than women (OR 0.80; 95%CI 0.73-0.89). Urban residence was associated with a higher likelihood of receiving care (OR 1.64; 95%CI 1.46-1.84), as was older age: 60-79 years old (OR 1.58; 95%CI 1.36-1.84) and those aged 80 years old or older (OR 1.95; 95%CI 1.67-2.27).

**Table 2.** Factors associated with receiving medical care before death from heart failure. Mexico, 2019 and 2023.

Characteristics	Categories	Adjusted OR	95%CI	***P*-value**
Year of death	2019	Ref.	-	-
	2023	1.22	1.10-1.36	<0.001
Gender	Female	Ref.	-	-
	Male	0.80	0.73-0.89	<0.001
Age (years)	<60	Ref.	-	-
	60-79	1.58	1.36-1.84	<0.001
	≥80	1.95	1.67-2.27	<0.001
Education	No schooling	Ref.	-	-
	Primary/secondary	1.51	1.35-1.69	<0.001
	High school or higher	2.05	1.70-2.47	<0.001
Marital status	Other	Ref.	-	-
	Married	1.31	1.16-1.48	<0.001
Health insurance coverage	Without coverage	Ref.	-	-
	With coverage	2.42	2.18-2.69	<0.001
Area of residence	Rural	Ref.	-	-
	Urban	1.64	1.46-1.84	<0.001
Region of the country	North	Ref.	-	-
	Central-North	1.93	1.65-2.27	<0.001
	Central	Ref.	2.64-3.64	<0.001
	South	1.91	1.61-2.26	<0.001
	Southeast	1.45	1.22-1.73	<0.001
Month of death	December - January	Ref.		
	Other months	1.27	1.13-1.43	<0.001
Place of death	Hospital/clinic	Ref.	-	-
	Home	0.37	0.33-0.43	<0.001
Year of death	2019	Ref.	-	-
	2023	1.22	1.10-1.36	<0.001
Interaction terms	Without health insurance and death at home	0.35	0.30-0.41	<0.001
	With health insurance and death at home	0.37	0.33-0.43	<0.001
	Without health insurance and death outside home	0.85	0.69-1.05	0.128

OR: odds ratio; 95%: 95% confidence interval. Multiple logistic regression model adjusted for gender, age, education, marital status, health insurance coverage, area of residence, region, place of death, and year.

Furthermore, a higher level of education was associated with a progressively increased likelihood of receiving medical care. Individuals with primary or secondary education had an OR of 1.51 (95%CI 1.35-1.69), while those with high school education or higher had an OR of 2.05 (95%CI 1.70-2.47). Marital status was also significantly associated with medical care, with a higher likelihood of receiving care among married individuals (OR 1.31; 95%CI 1.16-1.48).

**Figure 2.**
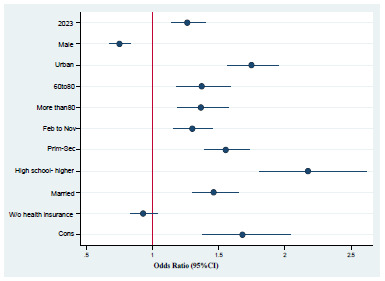
Forest plot of the adjusted odds ratio for receiving medical care before death from heart failure. Mexico, 2019 and 2023.

Regarding geographic region, compared with the northern region of the country, a higher probability of receiving medical attention was observed in the Central (OR 3.10), Central-North (OR 1.93), South (OR 1.91), and Southeast (OR 1.45) regions, with statistically significant differences (p<0.001) (Figure 2).

Finally, death occurring at home was associated with a significant reduction in the probability of having received medical care (OR 0.37; 95%CI 0.33-0.43) (Table 2).

### Interaction between health insurance and place of death

A significant reduction in the probability of receiving medical care was observed when individuals without health insurance died at home (OR 0.35; 95%CI 0.30-0.41). Similarly, individuals with health insurance who died at home had a lower probability of receiving care (OR 0.37; 95%CI 0.33-0.43). In contrast, lack of health insurance combined with death occurring outside the home was not significantly associated with reduced medical care (OR 0.85; 95%CI 0.69-1.05), suggesting that the effect of health insurance coverage is conditioned by the place of death (Table 2). The model demonstrated adequate discriminatory performance (AUC=0.713), with a sensitivity of 85.6%.

## DISCUSSION

This study identified the sociodemographic determinants associated with medical care prior to death from HF in Mexico during 2019 and 2023. The results showed that male and rural populations, as well as individuals with lower levels of education and those without health insurance, had a lower probability of receiving medical care prior to death, confirming the persistence of structural inequalities in access to healthcare services.

The finding that the probability of receiving medical care in 2023 was 22% higher than in 2019 may reflect an expansion of medical care following the COVID-19 pandemic. This result may be explained by the reorganization of the healthcare system and the reactivation of primary and hospital care services after the saturation observed in 2020 and 2021, when human and material resources were concentrated on emergency healthcare responses. Previous studies in Latin America and Mexico documented a considerable decrease in care for chronic diseases during the pandemic^22^ ^,^ ^23^, followed by an increase in consultations and hospitalizations during 2022-2023, which is consistent with the trend observed in this analysis.

In relation to gender, men showed a 20% lower probability of having received medical assistance prior to death, in accordance with international evidence indicating lower healthcare-seeking behavior among males^24^ ^,^ ^25^.

Men constitute a vulnerable group in access to healthcare services due to sociocultural factors that discourage seeking care, expressing symptoms, and adhering to treatment.

Several studies suggest that traditional patterns of masculinity are associated with lower risk perception, reduced use of preventive services, and higher mortality from preventable causes^26^.

In contrast, women tend to access medical services more frequently, partly due to greater contact with the healthcare system throughout their lives and a higher perception of risk, factors that positively influence timely medical care^27^ ^,^ ^28^.

The increased likelihood of receiving medical care among individuals aged 60 years old and older is consistent with international literature, which reports greater interaction with the healthcare system as age increases^29^.

This finding can be explained by a greater burden of comorbidities, a more acute perception of functional decline, and more frequent use of specialized healthcare services.

Depending on their place of residence, individuals living in urban areas were significantly more likely to have received medical attention prior to death. This difference reflects the unequal distribution of healthcare resources, which have historically been concentrated in the urban areas of the central and northern regions of the country.

In contrast, rural areas face infrastructure deficiencies, shortages of healthcare personnel, and limitations in transportation and access to specialized services^30^ ^,^ ^31^.

Furthermore, linguistic barriers can exacerbate inequalities, especially in indigenous communities, where more than 68 native languages are spoken in the Mexican Republic^32^. The absence of interpreters and the limited intercultural training of healthcare personnel restrict communication, reduce diagnostic quality, and discourage the search for timely medical attention^33^ ^,^ ^34^ **.**

Educational level acted as a positive determinant of the probability of receiving medical care.

Individuals with secondary education or higher were more likely to receive medical assistance, which may be attributed to better knowledge of the disease, higher health literacy, and improved economic conditions and access to healthcare services.

This finding is consistent with research indicating that education acts as a protective factor against mortality from HF and other chronic diseases^35^.

Similarly, being married was associated with a higher likelihood of having received medical care.

The presence of a partner or a close family network can facilitate the decision to seek healthcare services, as well as support healthcare-seeking behavior, therapeutic adherence, and continuity of care^36^.

One of the most significant findings was the interaction between health insurance coverage and place of death. Individuals without health insurance coverage who died at home were the least likely to have received medical attention, followed by those with health insurance coverage who also died at home. This result suggests the existence of structural barriers that transcend formal health insurance coverage, related to economic, geographic, or cultural factors.

Previous studies on avoidable mortality in Mexico have documented that lack of health coverage, higher levels of marginalization, and residence in rural areas increase the probability of dying without medical attention^37^.

The regression model demonstrated adequate discriminatory power, which reinforces the internal validity of the findings.

However, this study has limitations that should be considered.

The retrospective, cross-sectional study design prevents the establishment of causal relationships between variables.

Furthermore, the database used does not include detailed clinical information, such as time since diagnosis, ejection fraction, type of treatment, or the presence of additional comorbidities, which could influence the likelihood of receiving medical care.

Despite these limitations, the use of official sources with broad coverage and methodological validation ensures the reliability of the results. Taken together, the findings demonstrate that, although the Mexican healthcare system has shown progress in coverage and post-pandemic reorganization, significant inequities persist in access to medical services, particularly among rural populations, individuals with low levels of education, and those without health insurance. These results reinforce the need to strengthen effective universal coverage, expand medical care in rural and indigenous areas, and develop specific strategies targeting male and socially vulnerable populations to guarantee timely and equitable medical care.
