# Supplementary material for: Sociodemographic determinants of health care in heart failure mortality in Mexico, 019 and 2023
Source: Rev Bras Epidemiol. 2026 Apr 20;29:e260019. doi: 10.1590/1980-549720260019.2 (PMC13101396; doi:10.1590/1980-549720260019.2)
Supplement: Translation

# Translation: Determinantes sociodemográficos de los cuidados de salud en la mortalidad por insuficiencia Cardiaca en México, 2019 y 2023

**DOI:** 10.1590/1980-549720260019

**Authors:** Julio Cesar Campuzano, Jorge Martin Rodríguez, Pablo Enrique Chaparro, Diana Carolina Urrego, Anaid Hernández

**Affiliations:** I Instituto Nacional de Salud Pública de México - Cuernavaca, Morelos, México.; II Pontificia Universidad Javeriana, Instituto de Salud Pública - Bogotá D.C., Colombia.; III Observatorio Nacional de Salud - Bogotá D.C., Colombia.; IV Ministerio de Salud y Protección Social - Bogotá, Colombia.; V Universidad Latinoamericana - Cuernavaca, Morelos, México.

**Keywords:** Insuficiencia cardíaca, Mortalidad, Factores Socioeconómicos, Seguridad social, México

## Abstract

**Objetivo:**

Analizar los determinantes sociodemográficos asociados con la asistencia médica antes del fallecimiento por ICa en México, comparando 2019 y 2023.

**Métodos:**

Estudio transversal retrospectivo, con enfoque analítico, basado en registros nacionales de mortalidad de la Dirección General de Epidemiología (DGE). Se seleccionaron defunciones por ICa (CIE-10: I50.0-I50.9) correspondientes a 2019 y 2023, periodos representativos del contexto pre y pospandemia (COVID-19). Las variables incluidas fueron sexo, edad, escolaridad, estado civil, derechohabiencia, área de residencia, región, sitio de defunción y año de ocurrencia. Se aplicó regresión logística múltiple para estimar *odds ratio* (OR) con intervalos de confianza al 95% (IC95%).

**Resultados:**

En el periodo 2019-2023 se registraron 13.510 defunciones por ICa (6.077 en 2019 y 7.433 en 2023)**.** La probabilidad de recibir asistencia médica antes del fallecimiento fue 22% mayor en 2023 en comparación con 2019 (OR 1,22; IC95% 1,10-1,36). La asistencia fue más probable en personas de 80 años o más, con mayor escolaridad, residencia urbana y derechohabiencia. Ser Hombre, no contar con cobertura médica y fallecer en el hogar se asociaron con una menor probabilidad de recibir atención. La interacción entre derechohabiencia y sitio de defunción reveló una reducción de la atención médica en quienes no contaban con seguridad social y fallecieron en el hogar.

**Conclusiones:**

Persisten brechas sustanciales en el acceso a la atención médica asociadas con desigualdades estructurales y sociales. La recuperación de la cobertura posterior a la pandemia evidencia un fortalecimiento parcial del sistema de salud. Se recomienda fortalecer la atención primaria, ampliar la cobertura universal efectiva y desarrollar estrategias dirigidas a grupos vulnerables.

## INTRODUCCIÓN

La insuficiencia cardiaca (ICa) es un síndrome clínico caracterizado por síntomas y signos derivados de una anomalía estructural y/o funcional del corazón, que impide el adecuado bombeo de sangre para satisfacer las demandas metabólicas corporales^1^. Los principales factores de riesgo para desarrollar ICa incluyen la enfermedad cardíaca coronaria, la hipertensión arterial, la diabetes mellitus, la obesidad, los antecedentes familiares de enfermedad cardíaca, la enfermedad pulmonar crónica, la inflamación o infección crónica, los trastornos metabólicos, el tratamiento con agentes cardiotóxicos, el consumo de cocaína, la terapia con antraciclinas en oncología y el abuso de alcohol^2^.

Desde finales del siglo XX, la ICa ha sido reconocida como una epidemia debido al aumento exponencial de las hospitalizaciones atribuibles a esta causa^3^. En 2002, se estimaba que cerca de 6,5 millones de personas en Europa, 5 millones en Estados Unidos y 2,4 millones en Japón vivían con esta enfermedad^4^. Este incremento se ha relacionado con el envejecimiento poblacional^5^, asociándose con un mayor número de comorbilidades, discapacidad física y eventos adversos, lo que incrementa la demanda de atención médica y la mortalidad. En consecuencia, este síndrome se ha convertido en un problema de salud pública^6^.

Actualmente, las enfermedades cardiovasculares constituyen la segunda causa de mortalidad en el mundo, después del cáncer^7^, y en México representan la primera causa de muerte. De acuerdo con estimaciones del *Global Burden of Disease 2023*, más de 64 millones de personas viven con ICa a nivel mundial, con una prevalencia ajustada por edad de 831 casos por cada 100.000 habitantes^8^. La mayor carga se concentra en países de ingresos bajos y medios, donde la incidencia y la mortalidad permanecen elevadas debido al acceso limitado a los servicios de salud y al incremento de los factores de riesgo metabólicos. En contraste, los países de altos ingresos han mostrado una reducción sostenida de la mortalidad por ICa gracias a la expansión de terapias farmacológicas y al fortalecimiento de la atención primaria cardiovascular^9^.

No obstante, aunque los avances actuales en el tratamiento de la ICa han permitido mejorar la calidad de vida y la supervivencia de los pacientes, también se ha observado un incremento en las comorbilidades, la discapacidad y la dependencia funcional, lo que ha aumentado los costos para el sistema de salud. Este fenómeno se relaciona no solo con una mayor longevidad, sino también con el envejecimiento poblacional, reconocido como uno de los principales factores de riesgo para desarrollar ICa^10^.

A pesar de que las tecnologías actuales han permitido estabilizar o incluso reducir la incidencia de ICa en adultos mayores en algunas poblaciones, se han observado tendencias opuestas en personas jóvenes, posiblemente relacionadas con el aumento de otras patologías, como la obesidad^11^. Adicionalmente, se ha demostrado que características como la raza incrementan la incidencia de ICa antes de los 50 años, siendo más frecuente en personas de raza negra que en aquellas de raza blanca^12^.

Pese a la importancia de esta enfermedad, las estimaciones de la incidencia de ICa y sus tendencias temporales, incluso en países de altos ingresos, son escasas e inconsistentes^13^. En México, aunque no se dispone de datos precisos, se estima que 750.000 personas padecen ICa y que aproximadamente 75.000 nuevos casos se agregan cada año^14^. Este panorama se ve agravado por las desigualdades estructurales en el acceso a los servicios de salud.

El sistema de salud en México está conformado por un sector público y uno privado que operan de manera complementaria, aunque con marcadas desigualdades. El sector público incluye las instituciones de seguridad social - Instituto Mexicano del Seguro Social (IMSS), Instituto de Seguridad y Servicios Sociales de los Trabajadores del Estado (ISSSTE), Petróleos Mexicanos (PEMEX), Secretaría de la Defensa Nacional (SEDENA) y Secretaría de Marina (SEMAR) -, que brindan cobertura a aproximadamente el 52% de la población^15^. Por su parte, el sistema de atención a la población no asegurada, anteriormente Seguro Popular y, desde 2023, Instituto Mexicano de Seguridad Social Bienestar (IMSS Bienestar), cubre cerca del 35%, mientras que el 13% restante recurre a servicios privados o carece de acceso efectivo a atención médica^16^. Estas diferencias estructurales en la afiliación y la disponibilidad de recursos condicionan la equidad en la atención, especialmente en el manejo de enfermedades crónicas como la ICa^17^.

La magnitud de esta enfermedad, su impacto en la mortalidad y los elevados costos derivados de su atención resaltan su relevancia para la salud pública. En este contexto, el estudio de la mortalidad por ICa constituye una herramienta esencial para la planificación en salud, al permitir identificar desigualdades, priorizar recursos y formular políticas públicas basadas en evidencia^18^.

Por lo anterior, el objetivo de este estudio fue realizar un análisis comparativo de los determinantes sociodemográficos relacionados con la atención médica en personas que fallecieron por ICa en México durante los años de 2019 y 2023.

## MÉTODOS

### Diseño y fuentes de información

Se realizó un estudio transversal retrospectivo con un enfoque analítico, a partir de los registros individuales de defunciones de la Dirección General de Epidemiología (DGE) de México. Se seleccionaron las muertes cuya causa básica fue ICa, utilizando los códigos I50.0 a I50.9 de la Clasificación Internacional de Enfermedades, décima revisión (CIE-10).

Se eligieron los años 2019 y 2023 para representar los periodos previo y posterior a la pandemia por COVID-19, evitando el sesgo de los años intermedios (2020-2022), en los cuales se presentaron alteraciones en la atención médica y en la certificación de las defunciones.

### Lugar y área de estudio

El estudio se realizó a nivel nacional en México, abarcando las 32 entidades federativas. El país cuenta con una superficie de 1,96 millones de km² y una población estimada de 129 millones de habitantes en 2023. El Índice de Desarrollo Humano (IDH) nacional fue de 0,78 y el ingreso per cápita de 12.900 USD. Las regiones Centro y Norte concentran la mayor infraestructura sanitaria, mientras que el Sur y Sureste presentan rezagos históricos en cobertura y calidad de los servicios^19^.

### Fuente de datos

Los datos de mortalidad provinieron del Subsistema Epidemiológico y Estadístico de Defunciones (SEED), administrado por la DGE. Esta base se alimenta de los certificados de defunción procesados por el Registro Civil y el Instituto Nacional de Estadística y Geografía (INEGI), alcanzando una cobertura estimada del 98% a nivel nacional^20^. Estudios previos han documentado un subregistro inferior al 2%, con ligeras variaciones regionales, principalmente en entidades del sur del país.

### Variables y procesamiento de la información

La variable dependiente fue la “asistencia médica antes del fallecimiento” (sí/no). Las variables independientes incluyeron entidad federativa; región (norte, centro-norte, centro, sur y sureste); sexo; edad (menor de 60 años; 60 a 80 años; mayores de 80 años); mes de ocurrencia de la defunción (diciembre-enero y otros meses); escolaridad (sin escolaridad; primaria/secundaria; bachillerato y superiores); estado civil (casado/otros); derechohabiencia (con/sin); área de residencia (urbana/rural); año de ocurrencia de la muerte (2023/2019); habla lengua indígena (sí/no); sitio de ocurrencia de la muerte (hogar/otro lugar) y cohorte de nacimiento.

La variable dependiente “asistencia médica antes del fallecimiento” presentó una completitud del 97.6%, lo que garantiza la validez de los análisis realizados.

### Análisis de la información

Se calcularon tasas de mortalidad por cada 100.000 habitantes, específicas por grupos de edad y sexo, con sus respectivos intervalos de confianza al 95%. Las tasas se estimaron con base en las defunciones registradas por la DGE y las proyecciones poblacionales del Consejo Nacional de Población (CONAPO) y el INEGI.

### Análisis bivariado

Se exploraron las asociaciones entre la asistencia médica y las variables categóricas mediante la prueba de chi-cuadrado. Para variables continuas, como la edad, se aplicó la prueba *t* de *Student* para muestras independientes. Las variables con un valor de p<0,05 en el análisis bivariado fueron incluidas en el modelo de regresión logística múltiple.

### Análisis múltiple

Con base en los resultados del diseño transversal, se aplicó un modelo de regresión logística binaria para estimar las razones de momios (odds ratio - OR), los intervalos de confianza al 95% (IC95%) y los valores p. Las variables categóricas fueron transformadas en variables *dummy*, excluyendo una categoría de referencia para evitar colinealidad. Se exploró la interacción entre la derechohabiencia y el sitio de defunción mediante un término aditivo (dh#soc), con el fin de identificar efectos combinados sobre la probabilidad de haber recibido atención médica antes del fallecimiento.

El modelo se estimó mediante máxima verosimilitud utilizando la función *Logit* de la librería *statsmodels* en Python, complementada con *pandas* y *numpy*. Se empleó una estimación robusta de la matriz de varianzas-covarianzas (*cov_type*=’HC0’) para controlar la heterocedasticidad. Se reportaron las OR y sus IC95%.

### Consideraciones éticas

Este estudio fue clasificado como sin riesgo, al utilizar exclusivamente bases de datos secundarias, anonimizadas y de acceso público. De acuerdo con el Artículo 17 del Reglamento de la Ley General de Salud en Materia de Investigación para la Salud, los estudios que emplean datos de dominio público y anónimos se consideran sin riesgo y están exentos de revisión por un comité de ética^21^.

## Declaración de disponibilidad de datos:

El conjunto completo de datos que respalda los resultados de este estudio está disponible a pedido del autor correspondiente.

## RESULTADOS

En México, se registraron 13.510 defunciones por ICa entre 2019 y 2023, de las cuales 6.077 (45%) ocurrieron en 2019 y 7.433 (55%) en 2023. La tasa de mortalidad por 100.000 habitantes fue mayor en 2023 (5,61) que en 2019 (4,89), siendo ligeramente superior en mujeres que en hombres en ambos años (Figura 1).

**Figura 1.** Comparación de las tasas de mortalidad por insuficiencia cardiaca en hombres y mujeres. México, 2019 y 2023.

En los hombres, la tasa aumentó de 4,67 a 5,34 por 100.000 habitantes, mientras que en las mujeres pasó de 5,11 a 5,87, lo que refleja un incremento sostenido en ambos sexos, más pronunciado en los varones.

La Tabla 1 resume las características sociodemográficas de las personas fallecidas por ICa según la atención médica recibida antes del fallecimiento. En general, la mayoría de las personas recibió atención médica previa a la defunción. Las mujeres presentaron una mayor proporción de atención (86,7%) que los hombres (84,4%). En cuanto a la edad, el grupo de 80 años o más concentró la mayor cantidad de muertes (97%), y el 15,1% no recibió atención médica. Por región, el centro del país registró la proporción más alta de atención (92,2%), mientras que el norte y el sureste presentaron los valores más bajos (19,2 y 19,3% sin atención, respectivamente).

**Tabla 1.** Caracterización de los fallecidos por insuficiencia cardíaca según aspectos sociodemográficos y atención médica recibida. México, 2019 y 2023.

Variable	Categoría	Recibió atención médica (%)	No recibió atención médica (%)	IC95%
Sexo	Hombre	84,4	15,6	83,2-85,6
	Mujer	86,7	13,3	85,5-87,9
Edad (años)	<60	78,3	21,7	76,8-79,8
	60-79	85,2	14,8	84,1-86,3
	≥80	97,0	3,0	96,5-97,5
Escolaridad	Sin escolaridad	82,6	17,4	81,1-84,1
	Primaria/secundaria	88,9	11,1	87,8-90,0
	Bachillerato/superior	91,5	8,5	90,1-92,9
Estado civil	Casado	88,8	11,2	87,6-90,0
	Otros	84,2	15,8	82,9-85,5
Derechohabiencia	Con derechohabiencia	92,3	7,7	91,1-93,5
	Sin derechohabiencia	78,4	21,6	77,0-79,8
Mes de ocurrencia	Diciembre-enero	83,1	16,9	81,8-,84,5
	Otros meses	86,3	13,7	85,7-,86,9
Lugar de ocurrencia	Hogar	80,6	19,4	79,6-81,5
	Otros	90,9	9,1	90,2-91,6
Área de residencia	Urbana	87,9	12,1	86,5-89,3
	Rural	77,6	22,4	75,8-79,4
Región	Norte	80,8	19,2	78,9-82,7
	Centro-norte	89,1	10,9	87,8-90,4
	Centro	92,2	7,8	91,0-93,4
	Sur	86,7	13,3	85,2-88,2
	Sureste	80,7	19,3	78,9-82,5
Año de defunción	2019	84,5	15,5	83,2-85,8
	2023	86,8	13,2	85,6-88,0

IC95%: intervalo de confianza al 95%.

La atención médica fue más frecuente en zonas urbanas (87,9%) que en zonas rurales (77,6%), así como en personas casadas (88,8%) en comparación con las no casadas (84,2%) (Tabla 1).

El modelo de regresión logística múltiple (Tabla 2) mostró que las personas fallecidas en 2023 tuvieron 22% más probabilidad de haber recibido atención médica que aquellas fallecidas en 2019 (OR 1,22; IC95% 1,10-1,36). Los hombres presentaron un 20% menos probabilidad de recibir atención médica que las mujeres (OR 0,80; IC95% 0,73-0,89). La residencia urbana se asoció con una mayor probabilidad de atención (OR 1,64; IC95% 1,46-1,84), al igual que una mayor edad: 60-79 años (OR 1,58; IC95% 1,36-1,84) y 80 años o más (OR 1,95; IC95% 1,67-2,27).

**Tabla 2.** Factores asociados con la recepción de asistencia médica antes del fallecimiento por insuficiencia cardíaca. México, 2019 y 2023.

Variables	Categorías	OR ajustado	IC95%	***P*-valor**
Año de defunción	2019	Ref.	-	-
	2023	1,22	1,10-1,36	<0,001
Sexo	Mujer	Ref.	-	-
	Hombre	0,80	0,73-0,89	<0,001
Edad (años)	<60	Ref.	-	-
	60-79	1,58	1,36-1,84	<0,001
	≥80	1,95	1,67-2,27	<0,001
Escolaridad	Sin escolaridad	Ref.	-	-
	Primaria/secundaria	1,51	1,35-1,69	<0,001
	Bachillerato/superior	2,05	1,70-2,47	<0,001
Estado civil	Otros	Ref.	-	-
	Casado	1,31	1,16-1,48	<0,001
Derechohabiencia	Sin derechohabiencia	Ref.	-	-
	Con derechohabiencia	2,42	2,18-2,69	<0,001
Área de residencia	Rural	Ref.	-	-
	Urbana	1,64	1,46-1,84	<0,001
Región del país	Norte	Ref.	-	-
	Centro-norte	1,93	1,65-2,27	<0,001
	Centro	Ref.	2,64-3,64	<0,001
	Sur	1,91	1,61-2,26	<0,001
	Sureste	1,45	1,22-1,73	<0,001
Mes de ocurrencia	Diciembre - enero	Ref.		
	Otros meses	1,27	1,13-1,43	<0,001
Lugar de ocurrencia	Hospital / clínica	Ref.	-	-
	Hogar	0,37	0,33-0,43	<0,001
Año de defunción	2019	Ref.	-	-
	2023	1,22	1,10-1,36	<0,001
Interacción	Sin derechohabiencia y fallecimiento en hogar	0,35	0,30-0,41	<0,001
	Con derechohabiencia y fallecimiento en hogar	0,37	0,33-0,43	<0,001
	Sin derechohabiencia y fallecimiento fuera del hogar	0,85	0,69-1,05	0,128

OR: *odds ratio*; IC95%: Intervalo de confianza al 95%. Modelo de regresión logística múltiple ajustado por sexo, edad, escolaridad, estado civil, derechohabiencia, área de residencia, región, sitio de defunción y año.

Asimismo, un mayor nivel educativo se asoció con un incremento progresivo en la probabilidad de recibir atención médica. Las personas con educación primaria o secundaria presentaron un OR de 1,51 (IC95% 1,35-1,69), mientras que aquellas con estudios de bachillerato o nivel superior alcanzaron un OR de 2,05 (IC95% 1,70-2,47). El estado civil también mostró una asociación significativa, con una mayor probabilidad de atención entre las personas casadas (OR 1,31; IC95% 1,16-1,48).

En cuanto a la región, en comparación con el norte del país, se observó una mayor probabilidad de recibir atención médica en el centro (OR 3,10), centro-norte (OR 1,93), sur (OR 1,91) y sureste (OR 1,45), con diferencias estadísticamente significativas (p<0,001) (Figura 2).

**Figura 2.** Diagrama de bosque del ***odds ratio*** ajustado para la recepción de asistencia médica antes del fallecimiento por insuficiencia cardíaca. México, 2019 y 2023.

Finalmente, fallecer en el hogar se asoció con una reducción significativa en la probabilidad de haber recibido atención médica (OR 0,37; IC95% 0,33-0,43) (Tabla 2).

### Interacción entre derechohabiencia y sitio de defunción

Se observó una reducción significativa en la probabilidad de recibir atención médica cuando las personas sin derechohabiencia fallecieron en el hogar (OR 0,35; IC95% 0,30-0,41). Asimismo, las personas con derechohabiencia que fallecieron en casa presentaron una menor probabilidad de atención (OR 0,37; IC95% 0,33-0,43). En contraste, no tener derechohabiencia y fallecer fuera del hogar no se asoció de manera significativa con una menor atención médica (OR 0,85; IC95% 0,69-1,05), lo que sugiere que el efecto de la afiliación está condicionado por el lugar de la defunción (Tabla 2). El modelo mostró un adecuado desempeño discriminativo (AUC=0,713), con una sensibilidad del 85,6%.

## DISCUSIÓN

El presente estudio identificó los determinantes sociodemográficos asociados con la asistencia médica antes del fallecimiento por ICa en México durante los años 2019 y 2023. Los resultados mostraron que las poblaciones masculinas, rurales, con menor escolaridad y sin derechohabiencia presentaron una menor probabilidad de recibir atención médica antes de la defunción, lo que confirma la persistencia de desigualdades estructurales en el acceso a los servicios de salud.

El hallazgo de que en 2023 la probabilidad de recibir atención médica fue un 22% mayor que en 2019 puede reflejar una expansión de la asistencia médica posterior a la pandemia por COVID-19. Este resultado podría explicarse por la reorganización del sistema de salud y la reactivación de los servicios de atención primaria y hospitalaria tras la saturación observada en los años 2020 y 2021, cuando los recursos humanos y materiales se concentraron en la respuesta sanitaria de emergencia. Estudios previos en América Latina y México documentaron una disminución considerable en la atención de enfermedades crónicas durante la pandemia^22^ ^,^ ^23^, seguida de un aumento en consultas y hospitalizaciones en 2022-2023, lo que coincide con la tendencia observada en este análisis.

En relación con el sexo, los hombres mostraron una probabilidad un 20% menor de haber recibido asistencia médica antes del fallecimiento, en concordancia con la evidencia internacional que señala una menor búsqueda de atención médica entre varones^24^ ^,^ ^25^.

Los hombres constituyen un grupo vulnerable en el acceso a los servicios de salud debido a factores socioculturales que desincentivan la búsqueda de atención, la expresión de síntomas y la adherencia terapéutica.

Diversos estudios sugieren que los patrones tradicionales de masculinidad se asocian con una menor percepción de riesgo, un menor uso de servicios preventivos y una mayor mortalidad por causas evitables^26^.

Por el contrario, las mujeres suelen acceder con mayor frecuencia a los servicios médicos, en parte debido a un mayor contacto con el sistema sanitario a lo largo de la vida y a una percepción más elevada del riesgo, factores que influyen positivamente en la atención médica oportuna^27^ ^,^ ^28^.

La mayor probabilidad de recibir atención médica en personas de 60 años o más concuerda con la literatura internacional, que reporta una mayor interacción con el sistema de salud conforme aumenta la edad^29^.

Este hallazgo puede explicarse por una mayor carga de comorbilidades, una percepción más aguda del deterioro funcional y un uso más frecuente de servicios especializados.

Según el lugar de residencia, las personas que vivían en áreas urbanas presentaron una probabilidad significativamente mayor de haber recibido atención médica antes de la defunción. Esta diferencia refleja la distribución desigual de los recursos sanitarios, históricamente concentrados en las zonas urbanas del centro y norte del país.

En contraste, las zonas rurales enfrentan deficiencias en infraestructura, escasez de personal médico y limitaciones en el transporte o la disponibilidad de servicios especializados^30^ ^,^ ^31^.

Asimismo, las barreras lingüísticas pueden agravar las desigualdades, especialmente en comunidades indígenas, donde en la república mexicana se hablan más de 68 lenguas originarias^32^. La ausencia de intérpretes y la limitada capacitación intercultural del personal sanitario restringen la comunicación, reducen la calidad del diagnóstico y desincentivan la búsqueda de atención médica oportuna^33^ ^,^ ^34^.

El nivel educativo se comportó como un determinante positivo de la probabilidad de recibir atención médica.

Las personas con educación media o superior presentaron una mayor posibilidad de recibir asistencia, lo que puede atribuirse a un mejor conocimiento de la enfermedad, una mayor alfabetización en salud y mejores condiciones económicas y de acceso a los servicios.

Este hallazgo es consistente con investigaciones que señalan que la escolaridad actúa como un factor protector frente a la mortalidad por ICa y otras enfermedades crónicas^35^.

De igual forma, el estado civil casado se asoció con una mayor probabilidad de haber recibido atención médica.

La presencia de una pareja o de una red familiar cercana puede facilitar la decisión de acudir a los servicios de salud, así como el acompañamiento en la búsqueda de atención, la adherencia terapéutica y la continuidad de los cuidados^36^.

Uno de los hallazgos más relevantes fue la interacción entre derechohabiencia y sitio de defunción. Las personas sin derechohabiencia que fallecieron en el hogar presentaron la menor probabilidad de haber recibido atención médica, seguidas de aquellas con derechohabiencia que también murieron en casa. Este resultado sugiere la existencia de barreras estructurales que trascienden la afiliación formal, relacionadas con factores económicos, geográficos o culturales.

Estudios previos sobre mortalidad evitable en México han documentado que la falta de cobertura, el nivel de marginación y la residencia en zonas rurales incrementan la probabilidad de morir sin atención médica^37^.

El modelo de regresión mostró un adecuado poder discriminativo, lo que refuerza la validez interna de los hallazgos.

No obstante, este estudio presenta limitaciones que deben considerarse.

El diseño transversal retrospectivo impide establecer relaciones de causalidad entre las variables.

Además, la base de datos utilizada no incluye información clínica detallada, como el tiempo desde el diagnóstico, la fracción de eyección, el tipo de tratamiento o la presencia de comorbilidades adicionales, los cuales podrían influir en la probabilidad de recibir atención médica.

A pesar de estas limitaciones, el uso de fuentes oficiales con alta cobertura y validación metodológica permite garantizar la confiabilidad de los resultados. En conjunto, los hallazgos evidencian que, aunque el sistema de salud mexicano ha mostrado avances en cobertura y reorganización posterior a la pandemia, persisten brechas significativas de inequidad en el acceso a los servicios médicos, particularmente en poblaciones rurales, con baja escolaridad y sin derechohabiencia. Estos resultados refuerzan la necesidad de fortalecer la cobertura universal efectiva, ampliar la atención médica en zonas rurales e indígenas y desarrollar estrategias específicas dirigidas a poblaciones masculinas y socialmente vulnerables, con el fin de garantizar una atención médica oportuna y equitativa.
